# Role of *Trypanosoma cruzi* nucleoside diphosphate kinase 1 in DNA damage responses

**DOI:** 10.1590/0074-02760200019

**Published:** 2020-07-15

**Authors:** Chantal Reigada, Melisa Sayé, Fabio Di Girolamo, Edward A Valera-Vera, Claudio A Pereira, Mariana R Miranda

**Affiliations:** 1 Universidad de Buenos Aires, Facultad de Medicina, Instituto de Investigaciones Médicas A Lanari, Buenos Aires, Argentina; 2 Consejo Nacional de Investigaciones Científicas y Técnicas, Universidad de Buenos Aires, Instituto de Investigaciones Médicas, Laboratorio de Parasitología Molecular, Buenos Aires, Argentina

**Keywords:** Trypanosoma cruzi, NDPK, DNA repair, DNA damage, nucleoside diphosphate kinase

## Abstract

**BACKGROUND:**

NME23/NDPKs are well conserved proteins found in all living organisms. In addition to being nucleoside diphosphate kinases (NDPK), they are multifunctional enzymes involved in different processes such as DNA stability, gene regulation and DNA repair among others. TcNDPK1 is the canonical NDPK isoform present in *Trypanosoma cruzi*, which has nuclease activity and DNA-binding properties *in vitro*.

**OBJECTIVES:**

In the present study we explored the role of TcNDPK1 in DNA damage responses.

**METHODS:**

TcNDPK1 was expressed in mutant bacteria and yeasts and over-expressed in epimastigotes. Mutation frequencies, tolerance to genotoxic agents and activity of DNA repair enzymes were evaluated.

**FINDINGS:**

Bacteria decreased about 15-folds the spontaneous mutation rate and yeasts were more resistant to hydrogen peroxide and to UV radiation than controls. Parasites overexpressing TcNDPK1 were able to withstand genotoxic stresses caused by hydrogen peroxide, phleomycin and hidroxyurea. They also presented less genomic damage and augmented levels of poly(ADP)ribose and poly(ADP)ribose polymerase, an enzyme involved in DNA repair.

**MAIN CONCLUSION:**

These results strongly suggest a novel function for TcNDPK1; its involvement in the maintenance of parasite’s genome integrity.

Nucleoside diphosphate kinases (NDPKs) are ubiquitous and well conserved enzymes that catalyse the exchange of terminal phosphate between di- and tri-phosphate nucleosides.^1^ Their canonical and first discovered function is to maintain the intracellular pools of these nucleotides, however, NDPKs are considered multifunctional enzymes as they are involved in diverse physiological and pathological processes. Human NDPKs, comprised in the NME (NME23/NDPK) family, are the best studied NDPKs because of their crucial role as metastasis suppressors.^2^ Nuclear functions of NME proteins are currently being investigated in order to better understand the mechanisms underlying this function. For example, NME-H1 and NMEH-2 have been identified as potential transcription factors through their DNA-binding activities;^3^ and NME-H1 is also involved in the repair of DNA damages induced by UV and gamma radiation, bleomycin, cisplatin or etoposide in different cell lines, which is associated with an increased in its nuclear localisation.^4^ ^,^ ^5^ ^,^ ^6^

The DNA related functions of NDPKs are also being investigated in different organisms other than mammals, such as bacteria, plants, yeasts and protozoan parasites. A plenty of reports associate them to DNA processing activities because, like human ones, they are able to interact and cleave DNA.^7^ ^,^ ^8^ ^,^ ^9^ In addition, some NDPKs can be differentially regulated under gamma radiation exposure^10^ ^,^ ^11^ and can be required for repair of UV radiation and etoposide induced DNA damage.^12^

*Trypanosoma cruzi* is the protozoan parasite that causes Chagas’ disease. We have previously identified and characterised the *T. cruzi* NM23-H1 and NM23-H2 homologue, TcNDPK1, which is the only canonical NDPK present in the parasite.^13^ TcNDPK1 presents 54% and 64% identity with NM23-H1 and NM23-H2, respectively, and shares some common functional features of most of the canonical NDPKs such as unspecific *in vitro* nuclease activity,^7^ DNA-binding properties and cytosolic and peri-nuclear localisation.^14^ To date, there are few reports involving canonical NDPKs of trypasomatids in novel cellular processes; *Trypanosoma brucei* NDPK has been localised to the nucleus without any other functional characterisation^15^ and *Leishmania amazonensis* homologue was mainly described as a protein implicated in the cell infection by preventing ATP-mediated cytolysis of macrophages.^16^ Thus, in the present study we took the first steps to investigate the role of TcNDPK1 in DNA damage responses, finding that TcNDPK1 is involved in the maintenance of the parasite genome integrity.

## MATERIALS AND METHODS

*Parasites, yeasts and bacteria strains* - Epimastigotes of *T. cruzi*, Y strains (DTUII), were used in all the experiments. Parasites were cultured at 28ºC in plastic flasks (25 cm^2^) containing 5 mL of liver infusion tryptose (LIT) medium (started with 1 x 10^6^ cells mL^-1^) supplemented with 10% foetal calf serum, 100 U mL^-1^ penicillin, and 100 mg mL^-1^ streptomycin.^17^ The parasites were subcultured with passages each seven days. Genetic constructions and transgenic parasites were obtained as previously reported.^14^ Briefly, epimastigotes were transfected with a pTREX-TcNDPK1 (*TcNDPK1*-TritrypDB: TcCLB.508707.200) and a pTREX-GFP vector previously generated,^14^ cloned and maintained in LIT medium supplemented with G418 500 µg mL^-1^. Expression of TcNDPK1 was confirmed by enzymatic activity.

**Fig. 1:**
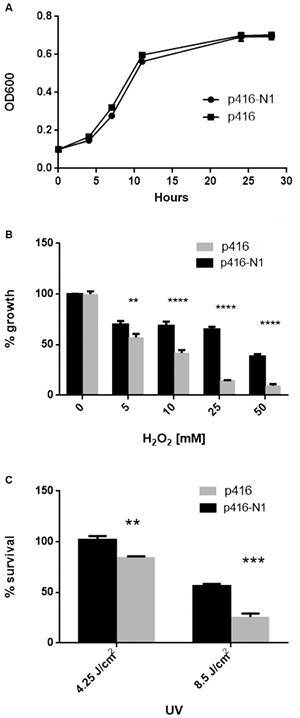
yeast treatment with genotoxic agents. Mutant yeasts lacking the endogenous *YNK1* but expressing the *TcNDPK1* gene were subjected to different genotoxic agents at the concentration indicated. (A) Growth curve of transfected yeasts measured at A_600nm_. (B) Percentage of yeast growth against H_2_O_2_ treatment. Yeasts were treated with different concentration of H_2_O_2_ and A_600nm_ was measured 24 h after treatment. Percentages were calculated considering 0 mM H_2_O_2_ as 100% C) Percentage of yeast survival against UVC radiation. Yeasts were loaded in petri dishes, subjected to 4.25 and 8.5 J/cm^2^ UVC radiation and incubated for 96 h. Percentages were calculated by counting colonies from radiated and non-radiated plates. ** p < 0.01; *** p < 0.001; **** p < 0.0001.

The mutant YNK1^-^ *Saccharomyces cerevisiae* strain (BY4741 MATa; *his3Δ1*; *leu2Δ0*; *met15Δ0*; *ura3Δ0, YKL067W*::*KanMX*) corresponds to the Euroscarf collection (http://www.euroscarf.de). Yeasts were transformed as previously described^18^ with an empty p416-GPD plasmid or a p416-TcNDPK1 plasmid constructed by the insertion of *TcNDPK1* gene from the pTREX-TcNDPK1 in the BamHI and XhoI recognition sites of p416-GPD. Transformed yeasts were grown in selective ura^-^ medium supplemented with histidine, leucine and methionine and checked by polymerase chain reaction (PCR) and enzymatic activity [Supplementary data **(Fig. 1)**].

**Fig. 2:**
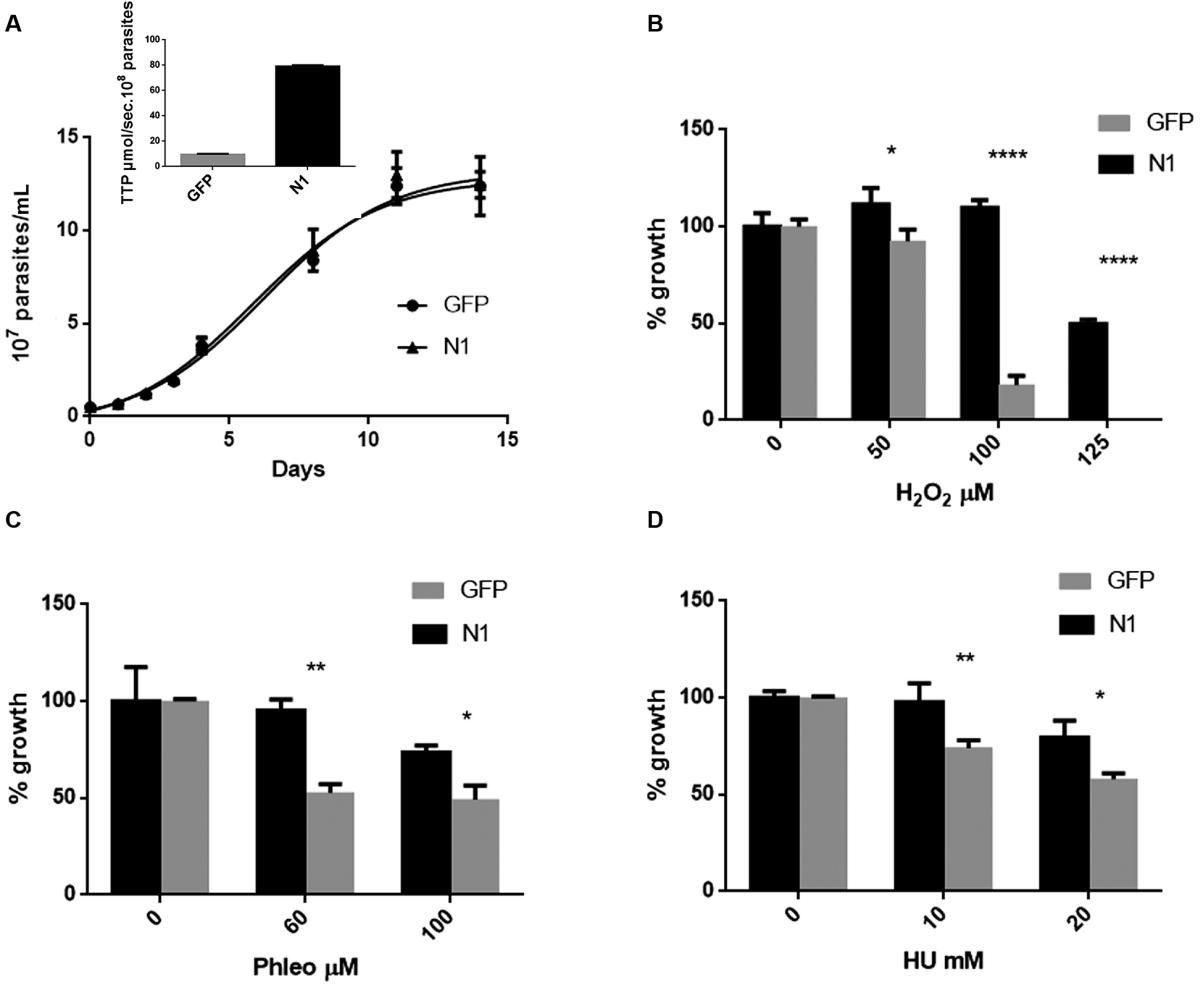
epimastigote treatment with genotoxic agents. Epimastigotes overexpressing the enzyme were cloned and (A) growth was monitored by counting under microscope. Inset: NDPK activity of overexpressing parasites (N1) and control parasites (GFP). Then, they were treated with different genotoxic agents at the concentrations indicated: (B) H_2_O_2_, (C) Phleo (phleomycin), (D) HU (hydroxyurea), and counted under microscope after 96 h (B and C) and 24 h (D) of treatment. Percentages were calculated considering 100% parasites without drug. GFP overexpressing parasites were used as control. * p < 0.05; ** p < 0.01; **** p < 0.0001.

Bacteria used in the study correspond to *Escherichia coli* BL21 (DE3) strain transformed with an empty pRSET-A and a pRSET-TcNDPK1 previously generated;^13^ and *E. coli* K-12 strain, *ndk* ^-^ mutant from the Keio collection,^19^ kindly provided by Dr Hirotada Mori (Nara Institute of Science and Technology, Japan) transformed with a pJexpress-404 bearing the gene of TcNDPK1 cloned in the BamHI and XhoI recognition sites (pJexpress-TcNDPK1). Both plasmids possess ampicillin (AMP) resistance cassette and K-12 *ndk* ^-^ mutant kanamycin (KAN) resistance cassette inserted in the *ndk* gene. All the constructions made for the study were checked by sequencing. Expression of TcNDPK1 was evaluated by standard protocols for his-tag protein purification and enzymatic activity [Supplementary data **(Fig. 2)**].

*Western blots* - Exponentially growing epimastigotes (5 x 10^7^ mL^-1^) were treated in LIT medium with hydrogen peroxide (H_2_O_2_) 3 mM and aliquots containing 1 x 10^7^ parasites were taken at different times (0, 5, 10, 20 and 30 min), immediately centrifuged and lysed in 15 µL of cracking buffer. Samples were cracked at 65ºC to avoid cleavage of PARP and totally loaded in a 15% acrylamide gel. Bovine serum albumin (BSA, 66 kDa) and ovalbumin (45 kDa) were used as molecular weight markers. The proteins were transferred to a polyvinylidene ﬂuoride (PVDF) membrane, blocked for an hour with 5% (w/v) non-fatty milk in T-PBS buffer (0.05% (v/v) Tween20, PBS 1x), incubated over night with rabbit anti-PARP antibodies (Santa Cruz Biotechnology) diluted 1:500 or anti-PAR reagent (MABE 1016, Millipore) diluted 1:500 in blocking buffer and finally incubated with HRP-conjugated anti-rabbit (Vector laboratories) antibodies diluted 1:5000 in blocking buffer. The membranes were revealed with enhanced chemiluminescence (ECL) reagent (Pierce). Parasites viability was assessed by direct light microscope observations and MTS colorimetric assays (CellTiter Aqueous One Solution Cell Proliferation Assay -Promega); they maintained shape and motility at 5, 10 and 20 min post-treatment, at 30 min motility was affected and viability decreased about 25%. For nuclear fraction analysis, 30 µg of protein was loaded and a dilution 1:2000 of a rabbit anti-TcHMGB^20^ serum was used.

*Immunofluorescences* - Immunofluorescences were carried out as previously reported, using TcNDPK1 overexpressing parasites (N1 parasites) and the same batch of polyclonal mouse anti-TcNDPK1 antibodies previously used by Pereira et al.^14^ Briefly, parasites treated with H_2_O_2_ 0.3 mM for 1 h or with phleomycin (Phleo) 150 µM for 4 h in LIT medium or without treatment (control) were settled on poly-L-lysine coated coverslips, fixed at room temperature for 20 min with 4% formaldehyde in PBS and permeabilised with cold methanol. After rehydration, the samples were blocked 10 min with 1% (w/v) PBS-BSA and incubated 45 min with mouse anti-N1 serum diluted 1:50. Then parasites were incubated 30 min with anti-mouse DyLight 488, diluted 1:500 in blocking buffer. Cells were mounted with Vectashield with DAPI (Vector Laboratories) and observed in an Olympus BX60 fluorescence microscope. Images were recorded with an Olympus XM10 camera. About 150 cells distributed in ten fields were analysed for each condition and percentages of parasites with enriched peri-nuclear localisation were calculated.

*Nuclear extraction and enzymatic activity* - Nuclear extraction was carried out according to Villanova et al.^21^ Briefly, 2 x 10^8^ wild-type (WT) parasites were lysed in lysis buffer A for 40 min [10 mM HEPES pH 7.9, 50 mM NaCl, 1 mM EDTA, 5 mM MgCl_2_, 0.05% (v/v) NP-40] and then glycerol was added to 5% (v/v). Nuclei were collected by centrifugation and washed twice with buffer B [10 mM HEPES pH7.9, 140 mM NaCl, 1 mM EDTA, 5 mM MgCl_2_, 5% (v/v) glycerol] and resuspended in buffer C [10 mM HEPES pH7.9, 400 mM NaCl, 0.1 mM EDTA, 5% (v/v) glycerol, 0.5 mM DTT]. Soluble, washes and nuclear fractions (S, W, N) were collected and used for activity and WB. NDPK and pyruvate kinase (PK) activity were measured recording NADH oxidation at A_340nm_ as previously reported.^13^ For evaluation of overexpressing parasites, soluble extract was obtained by suspending 2 x 10^8^ parasites in 100 mM Tris-HCl buffer pH 7.2 followed by five cycles of freezing and thawing.

*Genomic samples and electrophoresis* - 5 x 10^7^ mL^-1^ parasites were treated with H_2_O_2_ 3 mM in LIT medium and 1 mL-samples were collected at different times (0, 5, 10, 20 and 30 min), immediately centrifuged and resuspended in 0.3 mL of lysis buffer [10 mM Tris buffer pH 7.5, 100 mM EDTA, 0.1% (w/v) sodium dodecyl sulfate (SDS)]. These solutions were incubated with RNAse 30 min at 37ºC and then extracted with 0.3 mL of phenol. The aqueous phases were extracted with 0.3 mL of chloroform-isoamyl solution and genomic DNA was precipitated with isopropanol and recovered by centrifugation. Pellets were resuspended in 15 µL of TE buffer and quantified in a NanoDrop Nucleic Acid Quantification (Thermo Fisher). 200 ng of genomic DNA were loaded in an ethidium bromide-stained 1% agarose gel, electrophoresed at 50V for 2 h and visualised in a UVP gel Imaging and Documentation device (Bio-Rad).

*Genotoxic stresses* - 7.5 x 10^6^ exponentially growing epimastigotes were incubated in a 24 wells plate with different concentration of H_2_O_2_ (0, 50, 100 and 125 µM) in 300 µL of PBS at 28ºC for 2 h. Then, 1.2 mL of fresh LIT medium was added (5 x 10^6^ parasites mL^-1^ final) and parasites were incubated for other 96 h as previously reported.^18^ For Phleo and hydroxyurea (HU) treatments, same density of parasites were incubated in 500 µL of LIT medium with different concentrations of each genotoxic agents (Phleo: 0, 60 and 100 µg mL^-1^; HU: 0, 10 and 20 mM) for 96 h and 24 h respectively, as previously reported.^22^ After treatment, culture growth was monitored by parasite counting in a Neubauer chamber using an Olympus BX60 microscope.

In the case of *Saccharomyces cerevisiae*, experiments started from an overnight culture. H_2_O_2_ treatment was carried out by incubating 0.4 OD in 0.2 mL of PBS with different concentration of H_2_O_2_ (0, 5, 10, 25 and 50 mM) for 30 min. Then 2.8 mL of selective medium was added and incubated in agitation at 30ºC for 24 h. Yeast growth was measure by recording A_600nm_. For UV treatment, yeasts were diluted to 1 OD mL^-1^ (approximately, 1 x 10^7^ mL^-1^) and 50 µL of a 10^-4^ dilution was loaded into selective ura^-^ agar in a 6 cm petri plate and subjected to UVC radiation in a UVP gel Imaging and Documentation device (Bio-Rad) for 15 and 30 s. Then, plates were incubated at 30ºC for 96 h and colonies were counted. Survival percentages were obtained by taking as 100% the number of colonies without radiation.

*Rifampicin mutation frequency* - For K-12 strains, 12-20 cultures were started from a single colony in 3 mL 2YP-lactose broth (with 0.5 mM lactose added to allow basal transgene expression) and grown over night at 30ºC to avoid over saturation with the corresponding antibiotics (K12 *ndk* ^*-*^: 30 µg mL^-1^ KAN; K-12 *ndk* ^*-*^ /TcNDPK1: 30 µg mL^-1^ KAN and 100 µg mL^-1^ AMP). The number of rifampicin resistance (Rif^R^) mutants in each culture was determined by plating 1 mL of undiluted cultures on LB-Rif plates (100 µg mL^-1^ Rif, 30 µg mL^-1^ KAN and 100 µg mL^-1^ AMP when corresponding), and the total number of viable bacteria by plating 100 µL of a 10^-6^ dilution on LB plates. Frequencies were obtained by dividing the number of Rif^R^ mutants per total number of bacteria in each culture. Then, the average from each set of cultures was recorded. Sporadic jackpot cultures were removed from the analysis.

For BL21 strain, a similar approach was carried out, but 2YP-100 µg mL^-1^ AMP broth was used instead. Expression of transgene was due to the leaky expression of the pRSET system.^13^

*Data analysis* - All the experiments were made at least in triplicate (replicates) and results presented here are representative of three independent assays. When corresponding, data was analysed by t-test, one-way ANOVA or two-way ANOVA corrected by Sidack’s multiple comparison test with 95% confidence interval, using GraphPad Prism 6.01 for Windows.

Densitometries were carried out with the free available software ImageJ. Densities were normalised with bands of the corresponding Ponceau S red-stained membranes (loading control) or maxicircle bands and then referred to 0 min of N1 parasites.

## RESULTS

*Bacteria expressing TcNDPK1 have a lower spontaneous mutation frequency* - One experimental approach to identify possible proteins involved in DNA repair is by measuring the Rif^R^ mutation frequency of bacteria once incorporated the gene of interest,^23^ hence, the lesser mutation frequency obtained, the more genomic stability. In addition, strains lacking the endogenous *ndk* gene (*ndk* ^*-*^ *)* have been reported to have high mutation rates, thus being good for the evaluation of heterologous NDPKs.^24^ In order to determine if TcNDPK1 is involved in DNA stability processes we first measure its effect on bacterial spontaneous mutation frequency, assessed by determining the Rif^R^ incidence in transformed *E. coli* K12 mutants (K12 *ndk* ^*-*^ ) with a pJexpress plasmid bearing the *TcNDPK1* gene. The expression of *TcNDPK1* (K12 *ndk1* ^*-*^ /*TcNDPK1*) reduced the mutation frequency 15.68-folds respect to the control without the plasmid (K12 *ndk* ^*-*^ ), not reaching the frequency of K12 WT bacteria which was 40.09-folds lower than K12 *ndk* ^*-*^ and 2.56-folds lower than K12 *ndk1* ^*-*^ /*TcNDPK1*. Then, we also evaluated the expression of the *TcNDPK1* gene in *E. coli* BL21 bacteria using the pRSET vector, which produced a decrease of 5.2-folds in the spontaneous mutation frequency (Table).

**TABLE:** Bacterial mutation frequency

Strain	No. Rif^R^ per 10^8^ cells	Decreased folds
K-12 wild-type	1.08 (0.514-1.96) ****	40.09^*a*^
K-12 ndk ^-^	43.30 (18.7-85.8)	0
K-12 ndk ^-^/TcNDPK1	2.76 (1.53-4.77) ****	15.68^*a*^
BL21 pRSET	1.85 (1.21-3.6)	0
BL21 pRSET-TcNDPK1	0.352 (0.1-0.98)****	5.2^*b*^

The Rif^R^ mutation frequency was calculated in *Escherichia coli* K-12 bacteria, WT or mutants lacking the endogenous *ndk* gene, and in *E. coli* BL21 DE3, expressing or not TcNDPK1. The average of 12-20 independent assays is reported; in brackets are the ranges of frequencies obtained in each experiment. *a*: compared to WT; *b*: compared to BL21 pRSET; **** p < 0.0001.

*Yeasts expressing TcNDPK1 are more resistant to genotoxic agents* - As bacteria, yeasts provide an excellent model for the functional study of genes from other eukaryote organisms. Since the yeast homolog of *TcNDPK1* in *S. cerevisiae YNK1* is required for repairing UV radiation and etoposide induced DNA damage,^12^ then we tested if TcNDPK1 is able to complement a mutant yeast strain that lacks the endogenous gene *YNK1* (*YNK1* ^*-*^ ) when exposed to different DNA damage agents. It is well established that H_2_O_2_ and UV radiation cause DNA damage;^25^ H_2_O_2_ generates DNA oxidative damage causing single and double strand breaks (SSB and DSB, respectively), and UV produces DNA cross-linking lesions. For this purpose, mutant yeasts were transfected with the p416 vector bearing the *TcNDPK1* gene (p416-N1) or empty (p416) and survival capability was assessed after treatment with increasing concentrations of H_2_O_2_ (0, 5, 10, 25 and 50 mM) or exposures to UVC radiation (4.25 and 8.5 Jcm^-2^). Both yeast lines had similar growth kinetics but yeasts expressing the *T. cruzi* protein tolerated the different stresses better as is shown in Fig. 1; they were significantly more resistant than controls to all H_2_O_2_ concentrations assayed after 24 h of treatment (Fig. 1B) and to both UV exposures (Fig. 1C).

*Epimastigotes overexpressing TcNDPK1 are more tolerant to genotoxic agents* - Results obtained in bacteria and yeasts provided the first evidences of TcNDPK1 contribution to DNA stability, so the next step was to evaluate its role in *T. cruzi*. Protein overexpression is a common tool used in the study of protein function in this parasite. For this reason, we generated a TcNDPK1 overexpressing line of epimastigotes by transfecting them with a pTREX-TcNDPK1 plasmid (N1 parasites). Control parasites overexpressed the green fluorescent protein (GFP) with the same plasmid (GFP parasites). N1 parasites had an increase of 8.3-folds in the NDPK activity respect to the control, 79.1 ± 1.02 TTP µmol sec^-1^ per 10^8^ parasites and 9.5 ± 0.74 TTP µmol sec^-1^ per 10^8^ parasites for N1 and GFP, respectively (Fig. 2A, inset). In addition, the transgenic lines had similar culture growth and duplication rates, reaching same densities at stationary phase (Fig. 2A). Then, we evaluated the ability of these parasites to withstand different genotoxic stresses caused by H_2_O_2_ (0, 50, 100, 125 µM), Phleo (0, 60, 100 µM) and HU (0, 10, 20 mM) as reported by Gomes Passos Silva et al.^22^ Phleo produces DSB and HU causes replicative stress. N1 parasites were more tolerant to all these DNA damage inducing compounds as Fig. 2 shows. Notably, at 100 µM H_2_O_2_ 100% of N1 parasites survived to the treatment while only 20% of GFP parasites remained viable; in addition, 125 µM H_2_O_2_ was completely lethal to GFP parasites while 50% of N1 population persisted (Fig. 2B), measured 96 h after treatment. In contrast to H_2_O_2_ treatment, Phleo and HU stopped parasite replication without affecting their viability at the concentrations evaluated. Significant differences were observed after 96 h of treatment with 60 µg mL^-1^ and 100 µg mL^-1^ Phleo, reaching N1 parasites about 95% and 75% of growth respectively compared to 50% and 45% of control parasites (Fig. 2C). Regarding HU, N1 parasites were also significantly more resistant than GFP parasites to 24 h of replication stress, since N1 population was able to endure 24% more at 10 mM and 20 mM HU (Fig. 2D).

**Fig. 3:**
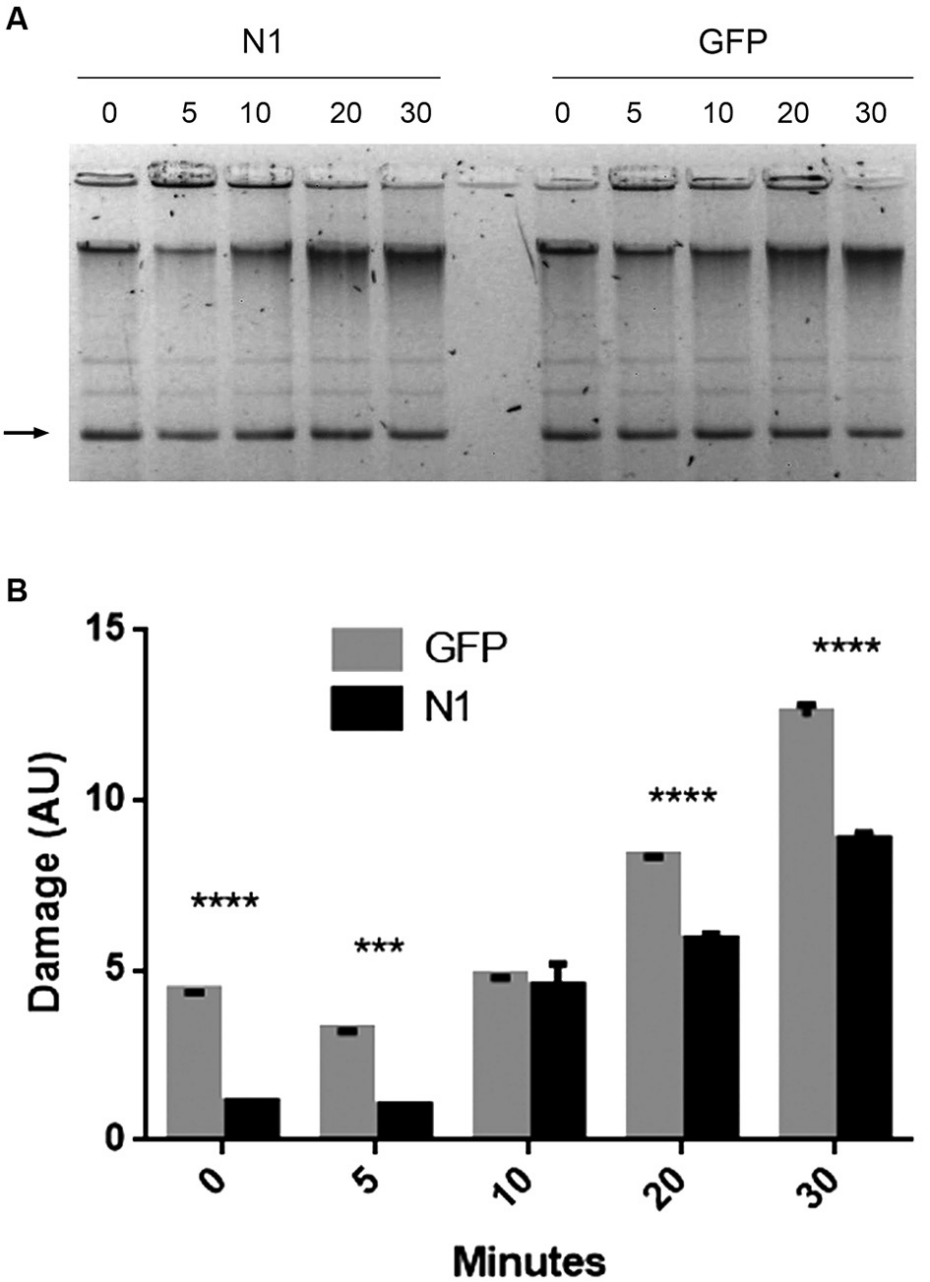
genomic DNA damage of epimastigotes. Parasites were treated with 3 mM H_2_O_2_ and (A) genomic DNA was isolated after 0, 5, 10, 20 and 30 min after treatment, loaded in an agarose gel and stained with ethidium bromide. Arrow points maxicircules used as loading control (B) Densitometry of A). GFP parasites were used as control. *** p < 0.001; **** p < 0.0001.

*Epimastigotes overexpressing TcNDPK1 have less genomic DNA damage and enhanced activity of TcPARP under H*_*2*_ *O*_*2*_ *treatment* - Our results showed that TcNDPK1 is involved in the response against different genotoxic agents, thus we decided to also evaluate its effect on genome stability. Previous works demonstrated that treatment with 0.03 to 3 mM H_2_O_2_ generates DNA damage at short times as 10 min.^26^ To assess the genomic damage caused by H_2_O_2_ in the transgenic lines, we first incubated parasites with 0.3 mM H_2_O_2_, extracted the genomic DNA at different times (0, 15, 30 and 60 min) and visualised it by agarose gel electrophoresis. At these conditions no DNA fragmentation was observed, hence parasites were exposed shorter times to 3 mM H_2_O_2_ (0, 5, 10, 20 and 30 min). Accordingly with the previous results, N1 parasites presented slightly less DNA damage than control parasites, even at 0 min, as can be seen in Fig. 3A-B. Furthermore, preliminary analysis of DNA damage by TUNEL showed similar results at 0 and 20 min of treatment [Supplementary data **(Fig. 3)**].

**Fig. 4:**
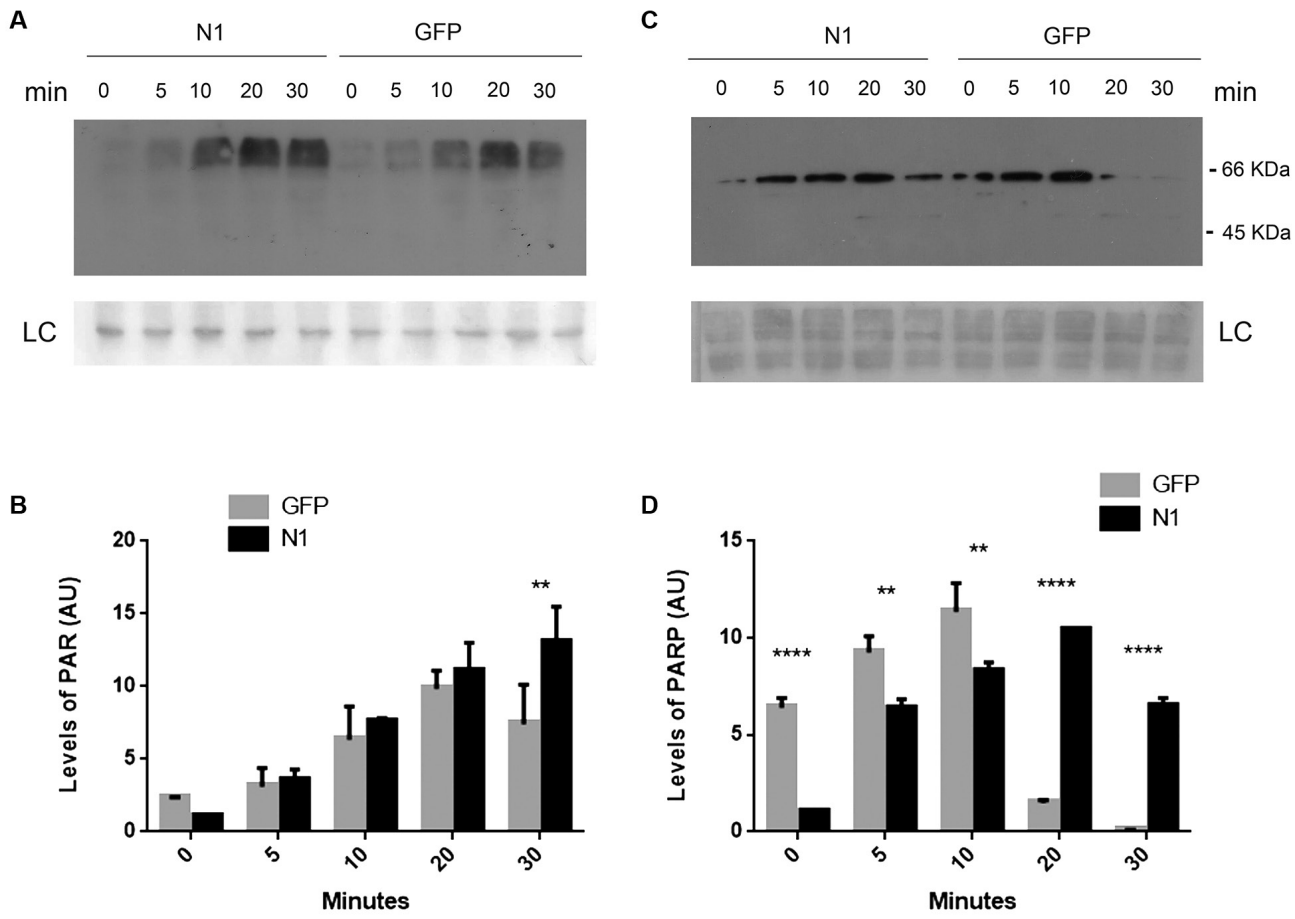
TcPARP activity. Parasites overexpressing TcNDPK1 were treated with 3 mM H_2_O_2_ and levels of (A) PAR and (C) TcPARP at 0, 5, 10, 20 and 30 min were measure by western blot. (B) and (D) are densitometries of (A) and (C) respectively. GFP parasites were used as control. LC: loading control; corresponding to Ponceau S red-stained membranes; ** p < 0.01; **** p < 0.0001.

We then explored possible mechanisms of action of TcNDPK1, since it participates in responses to DNA damaging agents that activate different repair pathways. One DNA break sensor that rapidly acts under diverse types of DNA damages is the Poly(ADP-ribose) polymerase (PARP), which catalyses the PARylation of nuclear proteins. *T. cruzi* possesses only one PARP of 65 KDa (TcPARP). At 3 mM H_2_O_2_ TcPARP is activated and translocates to the nucleus of the parasites.^26^ To further examine the activation of this protein in N1 and control populations, parasites were exposed to 3 mM H_2_O_2_ at different times (0, 5, 10, 20 and 30 min) and PARylation was evaluated by western blot (Fig. 4A). In both parasites the level of modified proteins increased with DNA damage up to 20 min, but, while N1 parasites maintained the elevated levels at 30 min, GFP control showed a 46% decrease at the same time (Fig. 4B). In accordance with PARylation, TcPARP levels augmented with exposure time (Fig. 4C). Notably, N1 parasites presented a continuous increase up to 20 min of treatment keeping the protein expression at high levels for 30 min, whereas in control parasites it showed an abrupt decrease being almost undetectable at the mentioned times (Fig. 4D). In addition, at 5 and 10 min, induction of TcPARP expression was more elevated in N1 parasites, being 6- to 10-fold higher respect to 0 min compared to 1.5- and 1.7-folds in GFP parasites (Fig. 4D).

**Fig. 5:**
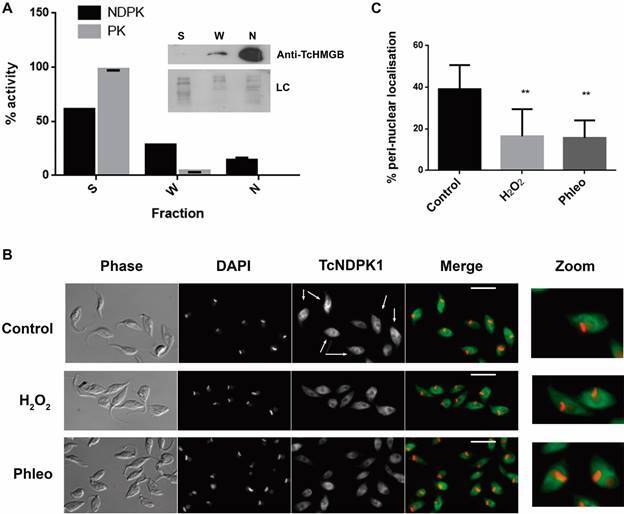
TcNDPK1 localisation. Parasites were subjected to nuclear extraction and immunofluorescence. (A) Analysis of fractions obtained from the nuclear extraction of WT epimastigotes; S: soluble; W: washes; N: nuclear. Pyruvate kinase (PK) activity was used as cytosolic marker. Inset: WB of the same fractions; TcHMGB was used as nuclear marker; LC: loading control. (B) Immunofluorescences of N1 parasites with anti-TcNDPK1 antibodies visualised with anti-mouse- DyLight 488. Parasites were no treated (control) or treated with 0.3 mM H_2_O_2_ for 1 h and with 150 µM Phleo for 4 h. Nuclei were stained with DAPI. Arrows point control parasites with a marked nuclear and peri-nuclear TcNDPK1 localisation. Scale bar: 5 µm. (C) Percentages of parasites with peri-nuclear enriched localisation of (B). ** p < 0.01.

*TcNDPK1 has nuclear localisation* - Nuclear localisation of proteins may have a specific function in this organelle. In a previous work, we determined that TcNDPK1 has a cytosolic localisation enriched mainly around the nucleus.^13^ ^,^ ^14^ In addition, the *T. brucei* orthologous NDPK was localised in the nucleus of the parasites.^15^ In order to analyse in detail the subcellular localisation of TcNDPK1, we evaluate the presence of NDPK activity in nuclei enriched fractions and carried out different immunofluorescence techniques. As shown in figure 5A, NDPK activity was detected not only in the cytosolic fraction but also in the nuclear fraction reaching about 15% of the total activity. Instead, 98% of PK activity (cytosolic marker)^13^ was present in the soluble fraction and was undetectable in the nuclear fraction. In accordance, a nuclear marker TcHMGB,^20^ was detected mainly in the nuclear fraction (Fig. 5A, inset). We also carried out immunofluorescences for TcNDPK1 and, as expected, about 40% of N1 population presented nuclear and peri-nuclear localisation, a pattern very similar to its *T. brucei* counterpart (Fig. 5B-C, for details, see^15^). Furthermore, Phleo and H_2_O_2_ treatment produced a slight delocalisation of TcNDPK1 out of the peri-nuclear region (Fig. 5B), reducing the number of parasites with enriched peri-nuclear localisation to 15% and 16% respectively (Fig. 5C).

## DISCUSSION

In the present study we provide the first evidences that *T. cruzi* NDPK1 is involved in DNA stability.

We determined that the heterologous expression of TcNDPK1 in different bacterial genetic backgrounds produced a decreased in their spontaneous mutation frequency; yeasts expressing TcNDPK1 were more tolerant to DNA damage induced by H_2_O_2_ and UV radiation; and overexpression of the enzyme in epimastigotes of *T. cruzi* generated parasites with improved ability to withstand to H_2_O_2_, Phleo and HU -induced DNA lesions. Furthermore, less genomic DNA damage could be observed in TcNDPK1 overexpressing parasites after H_2_O_2_ treatment. Interestingly, without stimulus GFP control parasites seem to have a basal DNA damage, probably due to the presence of SSB and DSB produced under physiological conditions. This basal damage is also reflected in greater levels of TcPARP and PAR, in contrast to N1 parasites that seemed to better overcome the same conditions.

Altogether, these results provide new insights in the role of TcNDPK1 in DNA damage responses. Supporting these evidences, Grynberg et al found that the expression of *TcNDPK1* is differentially regulated under gamma radiation exposure.^10^

H_2_O_2_, UV radiation, Phleo and HU generate distinct forms of DNA damage. H_2_O_2_ produces oxidative stress generating SSB and DSB, base modifications, apurinic/apirimidinic sites and DNA-protein cross-links;^27^ UV radiation produces intra-strand cross linking and pyrimidine photoproducts;^25^ Phleo is an antibiotic that binds to DNA and generates DSB and HU is an antimitotic inhibitor of the enzyme ribonucleotide reductase, that causes replicative stress.^22^ *T. cruzi* seems to have different ways to lead with DNA damage since most known DNA repair pathways are present in this organism.^27^ Probably, the genotoxic agents assessed in this study elicited different types of lesions and more than one repair pathway could be overlapping. One interesting enzyme that appears to be shared by the different pathways is PARP. PARP acts as a DNA damage sensor that promotes DNA repair at low levels of genotoxic stress, it is activated by binding to DNA-SSB and -DSB and modifies other target proteins with PAR chains. *T. cruzi* possesses only one PARP, TcPARP, which at the oxidative conditions assayed in the present study and under UVC radiation, translocates to the nucleus and increases the levels of PAR.^26^ In this scenario, to better understand a possible mechanism of action of TcNDPK1 in DNA damage responses, we evaluated the levels of PAR and TcPARP in the transgenic parasites. PARylation is a dynamic process, in *T. cruzi*, it has been reported that polymer formation increased continuously and then is completely degraded after 60 min H_2_O_2_ damage.^26^ This behaviour seems to be reproduced in our experiments, where PAR and TcPARP increase and then began to decrease at 30 min of H_2_O_2_ exposure in GFP parasites. On the contrary, N1 parasites have increased up regulation of TcPARP and PAR decay seems to be delayed, maintaining higher levels of both at 20 and 30 min. This suggests that TcPARP-mediated repair could be more efficient in N1 parasites because it is still active at longer times. These differences could explain the higher tolerance to genotoxic agents and less genomic damage observed, even when stimulus probably caused irreversible genomic damage to both *T. cruzi* populations (Fig. 3). These results suggest that, at least when dealing with DNA oxidative damage, TcNDPK1 response may involve TcPARP. Mechanisms linking TcNDPK1 and TcPARP are still unknown; however it could be hypothesised that TcNDPK1 acts upstream of TcPARP by regulating somehow its expression, thus eliciting an indirect role in DNA repair mechanisms. NDPKs are multifunctional enzymes and transcriptional regulation is another feature that contributes to this versatility.^3^ Furthermore, it is also possible that others TcNDPK1-mediated mechanisms operate overlapped and that they could work similarly in other organisms that lack PARP as bacteria and yeast. Actually, one well known mechanism relays on NDPK activity, since an imbalance in nucleotide pools can be mutagenic, thus contributing to genome integrity.^24^

The regulation of endogenous TcNDPK1 and the possible targets that it modulates under genomic damage remain to be elucidated. In this context, we preliminary explored NDPK activity of WT parasites treated 20 min with 3 mM H_2_O_2_ and interestingly, total NDPK activity of these parasites remained unaffected [Supplementary data **(Fig. 4)**]. Further experiments are needed to determine the underlying mechanisms in detail.

Numerous studies have confirmed a possible role in DNA repair for NN23-H1 and NM23-H2, human orthologous of TcNDPK1, through the base excision repair and nucleotide excision repair pathways (BER and NER, respectively) and also in DSB repair.^6^ ^,^ ^28^ Consistent with this role, NM23-H1 was reported to be peri-nuclear and nuclear and rapidly translocate to sites of DNA damage in the nucleus.^4^ ^,^ ^5^ The mechanism underlying nuclear translocation is poorly understood because these enzymes lack a canonical nuclear localisation signal, although passive diffusion is also expected due to their small molecular weight. We determine that TcNDPK1 possess cytosolic, peri-nuclear and nuclear localisation, a pattern very similar to the one reported for *T. brucei* NDPK.^15^ In addition, under oxidative damage and Phleo treatment, the marked peri-nuclear localisation is lost; however, we cannot affirm the enzyme has moved to the nucleus. Stimuli-dependent localisation of NDPKs has also been reported in other organisms,^29^ suggesting that TcNDPK1 delocalisation could be part of its regulation or mechanism of action.

The present report constitutes an exploratory study of the role of TcNDPK1 in DNA damage responses, and the results strongly suggest that TcNDPK1 is involved in genomic DNA maintenance. Further investigations will be needed to reveal in detail the extent to which TcNDPK1 participates in these processes. This knowledge will allow unravelling the intricate network of multiple functions of this enzyme.

## References

[1] Agarwal RP, Robison B, Parks RE (1978). Nucleoside diphosphokinase from human erythrocytes. Methods Enzymol.

[2] Steeg PS, Bevilacqua G, Kopper L, Thorgeirsson UP, Talmadge JE, Liotta LA (1988). Evidence for a novel gene associated with low tumor metastatic potential. J Natl Cancer Inst.

[3] Puts GS, Leonard MK, Pamidimukkala NV, Snyder DE, Kaetzel DM (2018). Nuclear functions of NME proteins. Lab Invest.

[4] Jarrett SG, Novak M, Dabernat S, Jean-Yves D, Mellon I, Zhang Q (2012). Metastasis suppressor NM23-H1 promotes repair of UV-induced DNA damage and suppresses UV-induced melanomagenesis. Cancer Res.

[5] Yoon JH, Singh P, Lee DH, Qiu J, Cai S, O'Connor TR (2005). Characterization of the 3' --&gt; 5' exonuclease activity found in human nucleoside diphosphate kinase 1 (NDK1) and several of its homologues. Biochemistry.

[6] Radic M, Sostar M, Weber I, Cetkovic H, Slade N, Herak Bosnar M (2020). The subcellular localization and oligomerization preferences of NME1/NME2 upon radiation-induced DNA damage. Int J Mol Sci.

[7] Miranda MR, Canepa GE, Bouvier LA, Pereira CA (2008). Trypanosoma cruzi nucleoside diphosphate kinase 1 (TcNDPK1) has a broad nuclease activity. Parasitology.

[8] Hammargren J, Salinas T, Marechal-Drouard L, Knorpp C (2007). The pea mitochondrial nucleoside diphosphate kinase cleaves DNA and RNA. FEBS Lett.

[9] Kumar P, Verma A, Saini AK (2005). Nucleoside diphosphate kinase from Mycobacterium tuberculosis cleaves single strand DNA within the human c-myc promoter in an enzyme-catalyzed reaction. Nucleic Acids Res.

[10] Grynberg P, Passos-Silva DG, Mourao MM, Hirata R, Macedo MM, Machado CR (2012). Trypanosoma cruzi gene expression in response to gamma radiation. PLoS One.

[11] Basu B, Apte SK (2012). Gamma radiation-induced proteome of Deinococcus radiodurans primarily targets DNA repair and oxidative stress alleviation. Mol Cell Proteomics.

[12] Yang M, Jarrett SG, Craven R, Kaetzel DM (2009). YNK1, the yeast homolog of human metastasis suppressor NM23, is required for repair of UV radiation- and etoposide-induced DNA damage. Mutat Res.

[13] Miranda MR, Canepa GE, Bouvier LA, Pereira CA (2008). Trypanosoma cruzi multiple nucleoside diphosphate kinase isoforms in a single cell. Exp Parasitol.

[14] Pereira CA, Reigada C, Saye M, Digirolamo FA, Miranda MR (2014). Cytosolic Trypanosoma cruzi nucleoside diphosphate kinase generates large granules that depend on its quaternary structure. Exp Parasitol.

[15] Hunger-Glaser I, Hemphill A, Shalaby T, Hanni M, Seebeck T (2000). Nucleoside diphosphate kinase of Trypanosoma brucei. Gene.

[16] Kolli BK, Kostal J, Zaborina O, Chakrabarty AM, Chang KP (2008). Leishmania-released nucleoside diphosphate kinase prevents ATP-mediated cytolysis of macrophages. Mol Biochem Parasitol.

[17] Camargo EP (1964). Growth and differentiation in Trypanosoma Cruzi I. Origin of metacyclic trypanosomes in liquid media. Rev Inst Med Trop São Paulo.

[18] Saye M, Miranda MR, di Girolamo F, Camara MM, Pereira CA (2014). Proline modulates the Trypanosoma cruzi resistance to reactive oxygen species and drugs through a novel D, L-proline transporter. PLoS One.

[19] Baba T, Ara T, Hasegawa M, Takai Y, Okumura Y, Baba M (2006). Construction of Escherichia coli K-12 in-frame, single-gene knockout mutants: the Keio collection.. Mol Syst Biol.

[20] Tavernelli LE, Motta MCM, Goncalves CS, Santos da Silva M.Elias MC.Alonso VL (2019). Overexpression of Trypanosoma cruzi high mobility group B protein (TcHMGB) alters the nuclear structure, impairs cytokinesis and reduces the parasite infectivity. Sci Rep.

[21] Villanova GV, Nardelli SC, Cribb P, Magdaleno A, Silber AM, Motta MCM (2009). Trypanosoma cruzi bromodomain factor 2 (BDF2) binds to acetylated histones and is accumulated after UV irradiation. Int J Parasitol.

[22] Silva DGP, Santos SS, Nardelli SC, Mendes IC, Freire ACG, Repoles BM (2018). The in vivo and in vitro roles of Trypanosoma cruzi Rad 51 in the repair of DNA double strand breaks and oxidative lesions. PLoS Negl Trop Dis.

[23] Aguiar PH, Furtado C, Repoles BM, Ribeiro GA, Mendes IC, Peloso EF (2013). Oxidative stress and DNA lesions the role of 8-oxoguanine lesions in Trypanosoma cruzi cell viability. PLoS Negl Trop Dis.

[24] Nordman J, Wright A (2008). The relationship between dNTP pool levels and mutagenesis in an Escherichia coli NDP kinase mutant. Proc Natl Acad Sci USA.

[25] Rastogi RP, Kumar AR, Tyagi MB, Sinha RP (2010). Molecular mechanisms of ultraviolet radiation-induced DNA damage and repair. J Nucleic Acids.

[26] Larrea SCV, Alonso GD, Schlesinger M, Torres HN, Flawia MM, Villamil SHF (2011). Poly(ADP-ribose) polymerase plays a differential role in DNA damage-response and cell death pathways in Trypanosoma cruzi. Int J Parasitol.

[27] Machado-Silva A, Cerqueira PG, Grazielle-Silva V, Gadelha FR, Peloso EF, Teixeira SMR (2016). How Trypanosoma cruzi deals with oxidative stress antioxidant defence and DNA repair pathways. Mutat Res Rev Mutat Res.

[28] Sheng Y, Xu M, Li C, Xiong Y, Yang Y, Kuang X (2018). Nm23-H1 is involved in the repair of ionizing radiation-induced DNA double-strand breaks in the A549 lung cancer cell line. BMC Cancer.

[29] Yoshida Y, Hasunuma K (2006). Light-dependent subcellular localization of nucleoside diphosphate kinase-1 in Neurospora crassa. FEMS Microbiol Lett.

