# Supplementary material for: Role of Trypanosoma cruzi nucleoside diphosphate kinase 1 in DNA damage responses
Source: Mem Inst Oswaldo Cruz. 2020 Jul 15;115:e200019. doi: 10.1590/0074-02760200019 (PMC7362669; doi:10.1590/0074-02760200019)
Supplement: Supplementary file 1 [file 1678-8060-mioc-115-e200019-s.pdf]

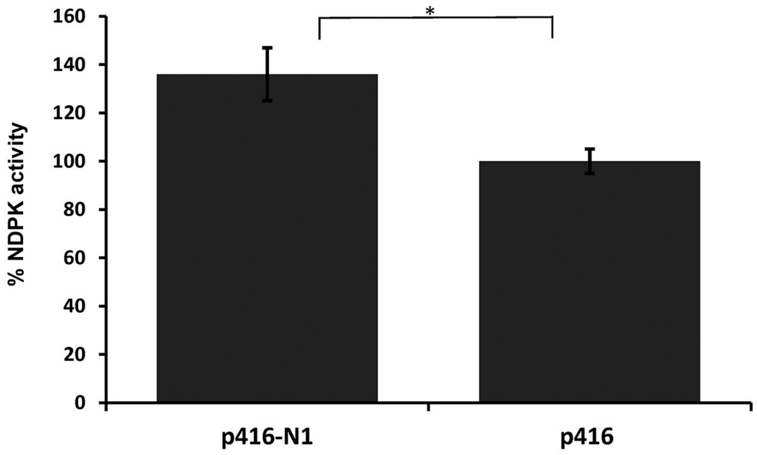

Fig. 1: transformed yeasts were grown overnight in selective ura^{-} medium. 3OD were collected, washed in PBS and suspended in 100 µL of 100 mM Tris-HCl pH 7.5. Then yeasts were frozen at -80ºC, thawed and disrupted by 15 cycles of 30 s in vortex with glass beads and 2 min in ice. After centrifugation, samples were used for NDPK activity as was detailed in MyM. Mutant yeasts bearing p416-N1 presented about 35% more activity than control yeasts. Null YNK1 mutant showed high basal NDPK activity, probably due to the presence of the yeast thymidylate kinase Cdc8 which has been reported to possess thymidylate-specific nucleoside diphosphate kinase activity in addition to thymidylate kinase activity (https://pubmed.ncbi.nlm.nih.gov/19540237). Furthermore, expression from p416-GPD vector is constitutive and low, thus expression of transgene and activity is expected to be also low (https://pubmed.ncbi.nlm.nih.gov/7737504/).

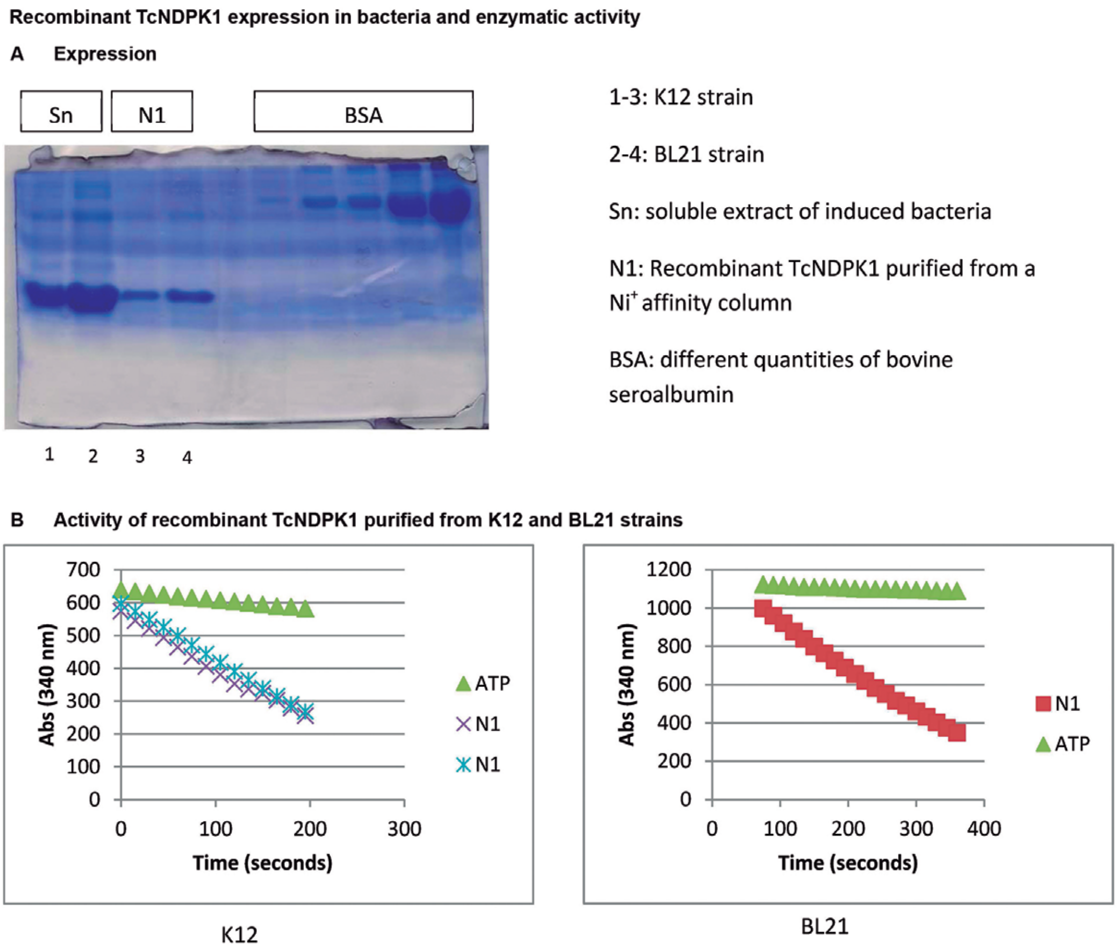

Fig. 2: his-tag TcNDPK1 (21 kDa) was purified as previously reported (https://pubmed.ncbi.nlm.nih.gov/18980701/). Samples electrophoresis was performed in a 15% acrylamide-Gel and stained with coomasie blue. BSA was used for quantification of samples. Activity was measured accordingly to MyM. Decrease in absorbance indicates presence of activity. 10 µL of each purified protein was added to the reaction mix. ATP: control with ATP and without dTDP.

CC BY

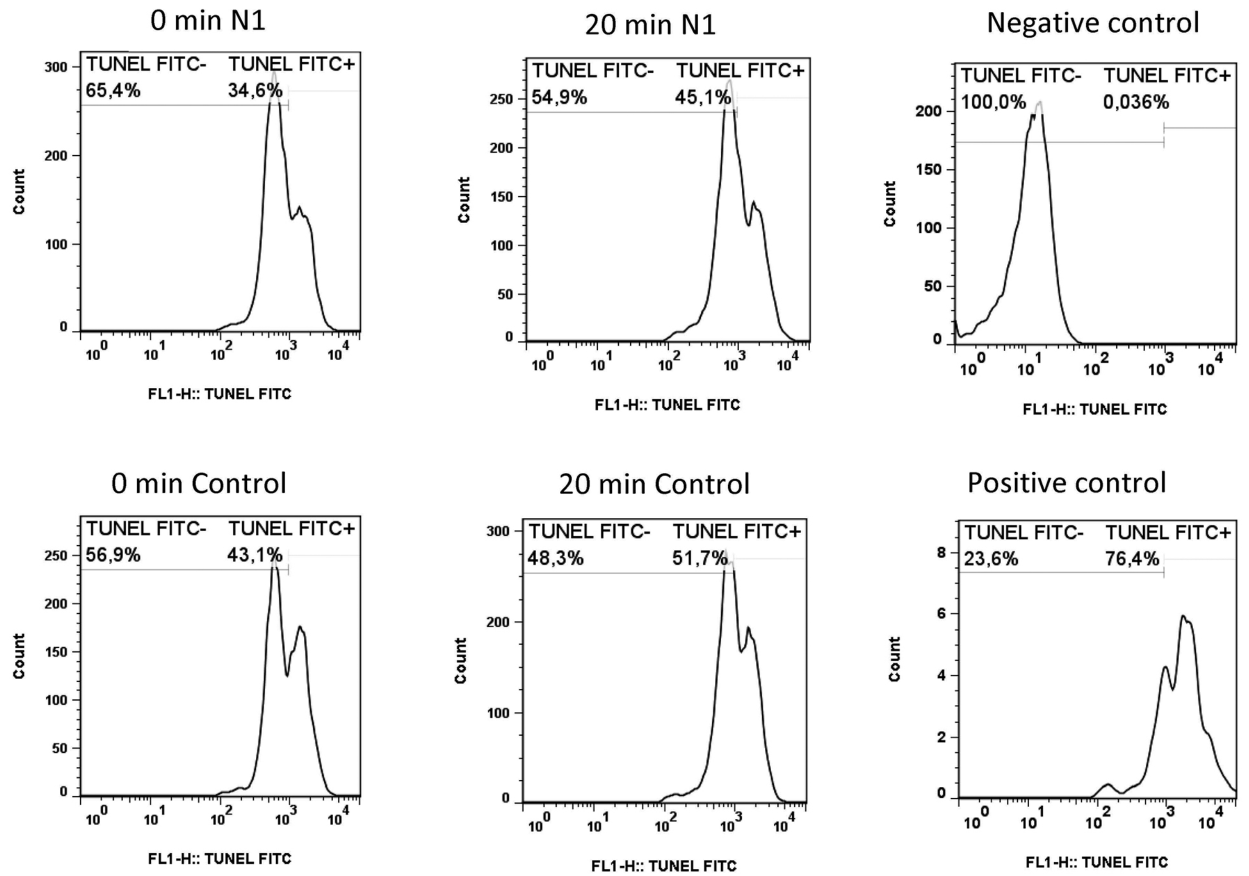

Fig. 3: for in situ DNA fragmentation detection by TUNEL, 5 × 10[7]/mL N1 and WT parasites were treated were treated 0 and 20 min with H_2O_2 3 mM in PBS, samples were immediately centrifuged and washed twice in PBS and fixed in 4% paraformaldehyde-PBS for 1 h at room temperature. Then parasites were washed and permeabilized 10 min in 0.1% (v/v) Triton X-100/PBS, blocked with 1 mM dATP 1% BSA-PBS-and stained according to the In Situ Cell Death Detection Kit (Roche) instruction manual. Control positive was done by incubating untreated WT parasites with 1 unit of DNAse in PBS and negative control was done without terminal deoxynucleotidyl transferase. Samples were kept in ice until analysis by flow cytometry (FACSAria II; BD Biosciences, Franklin Lakes, NJ) and results were analysed using the FlowJo software. Fluorescence intensity is proportional to the DNA damage. Basal intensities were high since in this conditions kintoplastid DNA is also stained in all the cells. WT parasites were used instead GFP parasites because GFP fluorescence interferes with TUNEL-FITC measurement.

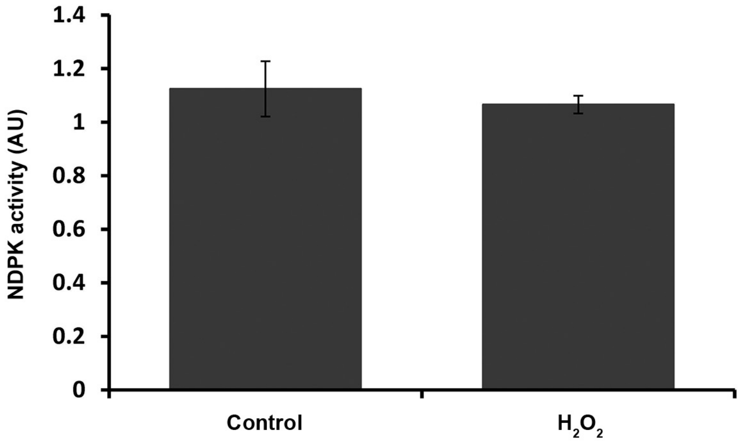

Fig. 4: 5 × 10[7]/mL WT parasites were treated with H_2O_2 3 mM in LIT medium for 20 min, washed and suspended in 100 mM Tris-HCl buffer pH 7.2. Extracts were obtained by five cycles of freezing and thawing. Enzymatic activity was carried out according to MyM.
